# Intermittent Theta-Burst Stimulation Transcranial Magnetic Stimulation Increases GABA in the Medial Prefrontal Cortex: A Preliminary Sham-Controlled Magnetic Resonance Spectroscopy Study in Acute Bipolar Depression

**DOI:** 10.3389/fpsyt.2021.665402

**Published:** 2021-05-11

**Authors:** Chad Diederichs, Marilena M. DeMayo, Jaeden Cole, Lakshmi N. Yatham, Ashley D. Harris, Alexander McGirr

**Affiliations:** ^1^Department of Psychiatry, University of Calgary, Calgary, AB, Canada; ^2^Hotchkiss Brain Institute, University of Calgary, Calgary, AB, Canada; ^3^Mathison Centre for Mental Health Research and Education, Calgary, AB, Canada; ^4^Department of Radiology, University of Calgary, Calgary, AB, Canada; ^5^Child and Adolescent Imaging Research Program, University of Calgary, Calgary, AB, Canada; ^6^Alberta Children's Hospital Research Institute, University of Calgary, Calgary, AB, Canada; ^7^Department of Psychiatry, University of British Columbia, Vancouver, BC, Canada

**Keywords:** repetitive transcranial magnetic stimulation, intermittent theta burst stimulation, bipolar disorder, bipolar depression, magnetic resonance spectroscopy, gamma-aminobutyric acid

## Abstract

**Background:** Magnetic resonance spectroscopy (MRS) has been used to identify gamma-aminobutyric acid (GABA) alterations in mood disorders, particularly in the medial prefrontal cortex (mPFC) where decreased concentrations have been associated with anhedonia. In major depressive disorder (MDD), prior work suggests that repetitive transcranial magnetic stimulation (rTMS) increases mPFC GABA concentrations proportional to antidepressant response. To our knowledge, this has not been examined in acute bipolar depression.

**Methods:** As part of a multicentre 4-week randomized, double-blind, sham-controlled trial using intermittent theta-burst stimulation (iTBS) of the left dorsolateral prefrontal cortex (DLPFC) in individuals with acute bipolar depression, we quantified mPFC GABA and Glx (glutamate+glutamine) concentrations using a 3T MRS scan at baseline and after the intervention. Depressive symptoms were measured using the Montgomery-Asberg Depression Rating Scale (MADRS) and the Hamilton Depression Rating Scale-17 (HRDS-17), and anhedonia was measured using the Snaith-Hamilton Pleasure Scale (SHAPS).

**Results:** The trial was terminated for futility and magnetic resonance spectroscopy data was acquired for 18 participants. At baseline, there were no associations between GABA or Glx concentrations and anhedonia, however GABA was negative correlated with depressive symptom severity on the HRDS-17. Compared to the sham-iTBS group, participants receiving active-iTBS had a significant increase in mPFC GABA concentrations. This was unrelated to antidepressant outcomes or improvements in anhedonia.

**Conclusion:** Our data suggests that iTBS targeting the DLPFC is associated with physiological changes in the mPFC. In acute bipolar depression, our preliminary data suggests that mPFC GABA is dissociated from antidepressant iTBS treatment outcomes and anhedonia.

## Introduction

Bipolar disorder (BD) is a leading cause of global disability that is defined by episodes of mania or hypomania (1). Yet, patients with BD type I spend as much as 70% of the time of the symptomatic periods experiencing syndromic or sub-syndromic depressive symptoms, while this proportion is over 80% in patients with BD type II (2). There are currently only four treatments that have been approved by the US Food and Drug Administration for the treatment of acute bipolar depression, namely olanzapine and fluoxetine combination, quetiapine, lurasidone, and caripirazine, and limited alternatives for those who do not respond to or tolerate these treatments (3).

Non-invasive neurostimulation techniques, such as transcranial magnetic stimulation (TMS), are efficacious in the treatment of Major Depressive Disorder (MDD). Accordingly, they are often considered in acute bipolar depression, however the literature in BD is small (4, 5). Newer TMS protocols, such as intermittent theta-burst stimulation (iTBS), are efficacious in MDD (6, 7) but have not demonstrated efficacy in BD to date (8, 9). Given lack of efficacy in BD, this may provide an opportunity to dissociate physiological effects that are and are not treatment mechanisms.

A growing body of research highlights alterations in brain gamma-aminobutyric acid (GABA) and glutamate systems in mood disorders (10, 11). ^1^H-MRS studies report decreases in cortical GABA in unipolar depression that correlate inversely with glutamate (12–15). Lower GABA levels have been repeatedly seen in the medial prefrontal cortex (mPFC) in depression, and this has been associated with anhedonia (16–18). Importantly, mPFC GABA levels increase with successful pharmacological treatment of MDD (19), as well as with repetitive transcranial magnetic stimulation (rTMS) treatment (20).

Several lines of evidence highlight the importance of the mPFC, and the anterior cingulate cortex more specifically, in the therapeutic effects of rTMS when the target is the dorsolateral prefrontal cortex (DLPFC). Indeed, a single treatment to the left DLPFC is associated with sustained changes in fMRI BOLD response and the GABA/Glx ratio in the anterior cingulate (21). Hyperactivity of the anterior cingulate predicts successful rTMS treatment (22), and clinical response to rTMS is associated with the degree of anticorrelated activity between the precise DLPFC target and the anterior cingulate cortex (23–25). In light of these findings, GABA concentrations in the medial prefrontal cortex (mPFC) may represent a physiological marker associated with large scale network changes in depression (26) that are amenable to rTMS.

To our knowledge, mPFC GABA concentrations during rTMS treatment in acute bipolar depression have not been measured. Therefore, in a randomized sham-controlled trial that examined the efficacy of iTBS in comparison to sham-iTBS (9), we used magnetic resonance spectroscopy (MRS) to assess mPFC GABA in the study patients. We hypothesized that mPFC GABA would be related to anhedonia, would increase with active-iTBS but not sham-iTBS, and that the change would be related to antidepressant response.

## Methods

We conducted a double-blind randomized controlled study in two Canadian centers (the University of British Columbia and University of Calgary) between October 2016 and March 2020. The study was registered with ClinicalTrials.gov (NCT02749006) and was approved by the clinical ethical review boards of the University of British Columbia and conjoint health ethical review board of the University of Calgary. The University of Calgary site, but not the University of British Columbia site, acquired GABA-edited MEGA-PRESS data in the mPFC.

The primary outcomes are described elsewhere (9). Briefly, the trial was terminated for futility after 37 participants were randomized due to the absence of an efficacy signal and overall low rates of clinical response (15.7% sham-iTBS vs. 16.6% active-iTBS). This low rate of clinical response was also observed after a 4-week open-label continuation for those that had not responded in the double-blind phase (23.8%).

### Participants

Participants were recruited by referral or by online and community advertisements. Participants provided written informed consent. Eligibility criteria included: males and females 18–70 years of age with a primary diagnosis of Bipolar I Disorder or Bipolar II (BD1 or BD2), experiencing a major depressive episode with ≥15 on the 17-item Hamilton Depression Rating Scale (HRDS-17) (27), and having failed to achieve clinical response with CANMAT first-line recommended treatments (lithium, lamotrigine, quetiapine, lurasidone with or without concurrent lithium or valproate) for an acute major depressive episode (3). Participants were required to have been on a stable pharmacological regimen for 2 weeks prior to screening that had to include a mood stabilizer (lithium >0.6 mEq/L or valproate >350 mM/L), an atypical antipsychotic, or a combination of a mood stabilizer and an atypical antipsychotic. For participants with BD2 only, lamotrigine monotherapy was acceptable if the dose was >100 mg daily.

Exclusion criteria were acute suicidality; current psychosis; a substance use disorder within the last 3 months; seizures; a pacemaker or metallic implant; an unstable medical condition; previous TMS; current use of more than three antipsychotic agents; failed response to ECT in current episode; psychotherapy initiated within the last 3 months; a psychiatric condition other than BD that was deemed to be primary.

### Treatment Protocol and Randomization

We generated three 1:1 randomization sequences for patients treated with (a) mood stabilizers, (b) atypical antipsychotics, or (c) a combination of mood stabilizer and antipsychotics to ensure that randomization was stratified according to current pharmacotherapy. Eligible patients were randomized with allocation concealment. Patients and clinical evaluators remained blind to their treatment condition. We utilized a MagPro X100 stimulator (MagVenture, Denmark), and a COOL-B70 or MCF-P-B70 placebo coil in conjunction with participant anatomical MRIs and neuronavigation (Visor2, ANT Neuro, the Netherlands). Resting motor threshold (rMT) was determined using electromyographic (EMG) electrodes placed over the first dorsal interosseous muscle, with threshold determined by the stimulus intensity required to elicit 5/10 EMG responses >50 μV.

Patients were randomly allocated to either active or sham iTBS, consisting of a total of 600 pulses per session. Pulses were delivered as triplets at 50 Hz repeated at 5 Hz (2 on 8 s off) and at 120% rMT. These parameters reflect the stimulus parameters that have been shown to be non-inferior to gold standard high-frequency stimulation in a large single blind study (28). We targeted the participants' left DLPFC using neuronavigation (Visor 2, ANT Neuro) using the T1-weighted MRI acquired at baseline. For participants who could not tolerate the MRI, we used the BeamF3 method which was then registered to a template MRI to permit reliable targeting for the duration of the study (29).

### Clinical Measures and Self-Reports

Participants were assessed by an independent evaluator (AM) blinded to treatment conditions. The diagnosis of BD was confirmed with the MINI International Neuropsychiatric Interview 7.0. Clinician rated instruments included the Hamilton Rating Depression Scale 17-item (HRDS-17), the Montgomery-Asberg Depression Rating Scale (MADRS) (30), the Young Mania Rating Scale (YMRS) (31), and the Clinical Global Impression (CGI) subscales for severity and improvement (32) at baseline, after 2 weeks, and after 4 weeks of double-blind treatment. Clinical response was defined as a reduction of 50% or more in MADRS score, and clinical remission was defined as a MADRS score of 12 or less. To measure anhedonia, participants completed the Snaith-Hamilton Pleasure Scale (SHAPS) (33) at baseline and at the conclusion of the double-blind phase.

### GABA-MRS

**Figure 1:**
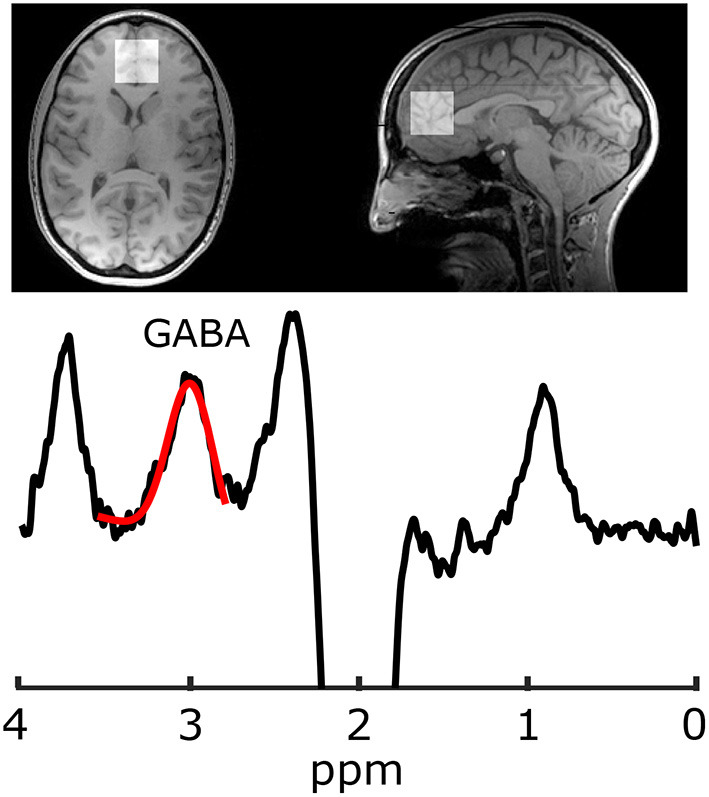
Representative T1 weighted image with mPFC voxel placement and GABA-edited MEGAPRESS.

Imaging was performed at 3 T (MR750, General Electric Healthcare, USA). Because the GABA peaks are overlapped by more abundant metabolite peaks, advanced spectroscopy methods are required for its accurate quantification; the most common being J-difference editing (34). For voxel localization and subsequent segmentation, first a T1-weighted acquisition was performed (TR/TE = 8.3/3.2 ms, 1mm isotropic voxels, aligned to the AC-PC line). Gamma-aminobutyric acid data were acquired from a 30 × 30 × 30 mm^3^ voxel in the mPFC using a GABA-edited MEGA-PRESS acquisition (TR/TE = 2 s/68 ms, 320 averages, 14 ms editing pulses alternating between 1.9 and 7.46 ppm every two averages, and eight unsuppressed averages for metabolite quantification). Voxels were then placed parallel to the AC-PC line anterior to the genu of the corpus callosum (Figure 1). Gamma-aminobutyric acid and the co-edited Glx (glutamate+glutamine) peak were analyzed in Gannet (35) including voxel localization and segmentation with SPM12, tissue correction for tissue specific T1 and T2 relaxation, and water visibilities (36, 37). Gamma-aminobutyric acid data also included tissue correction for concentration differences of GABA in white and gray matter, assuming the concentration of GABA in gray matter is twice that of white matter (38).

Data quality was visually assessed, which included assessing the GABA peak and resolution of subtraction artifact following spectral alignment, confirming the stability of water frequency (indicative of movement or scanner drift), confirming the quality of creatine alignment and fit of the spectrum and residuals. The included MEGA-PRESS acquisitions had a GABA fit error of 7.74 ± 3.28, full-width half-maximum of 22.03 ± 4.48 Hz, and signal to noise ratio of 11.97 ± 3.05.

### Voxel Placement Consistency

The T1-weighted image from the follow-up session was registered to the baseline acquisition [FSL's FLIRT (39, 40)] and the transformation matrix for that registration was then applied to the follow-up voxel such that both voxels for each subject could be visualized in the same space to ensure voxel placement consistency. In addition to visual confirmation of voxel placement consistency across sessions, the dice coefficient of the voxel overlap was calculated. The average dice coefficient was 72% (standard deviation = 10%).

### Statistical Analysis

Statistical analysis was performed in SPSS (IBM SPSS Statistics Version 26, IBM). Data normality was confirmed using the Shapiro-Wilk test and outliers were identified using Tukey's Fences (41). Cross-sectional analyses of continuous data were performed using Student's *t*-test or Mann Whitney U Test. Chi-square was used for dichotomized variables. One way ANOVA and linear regression was used to explore relationships between mPFC GABA and clinical characteristics. Change in mPFC GABA was quantified with repeated measures ANOVA. Two tailed significance was set at α < 0.05.

## Results

**Figure 2:**
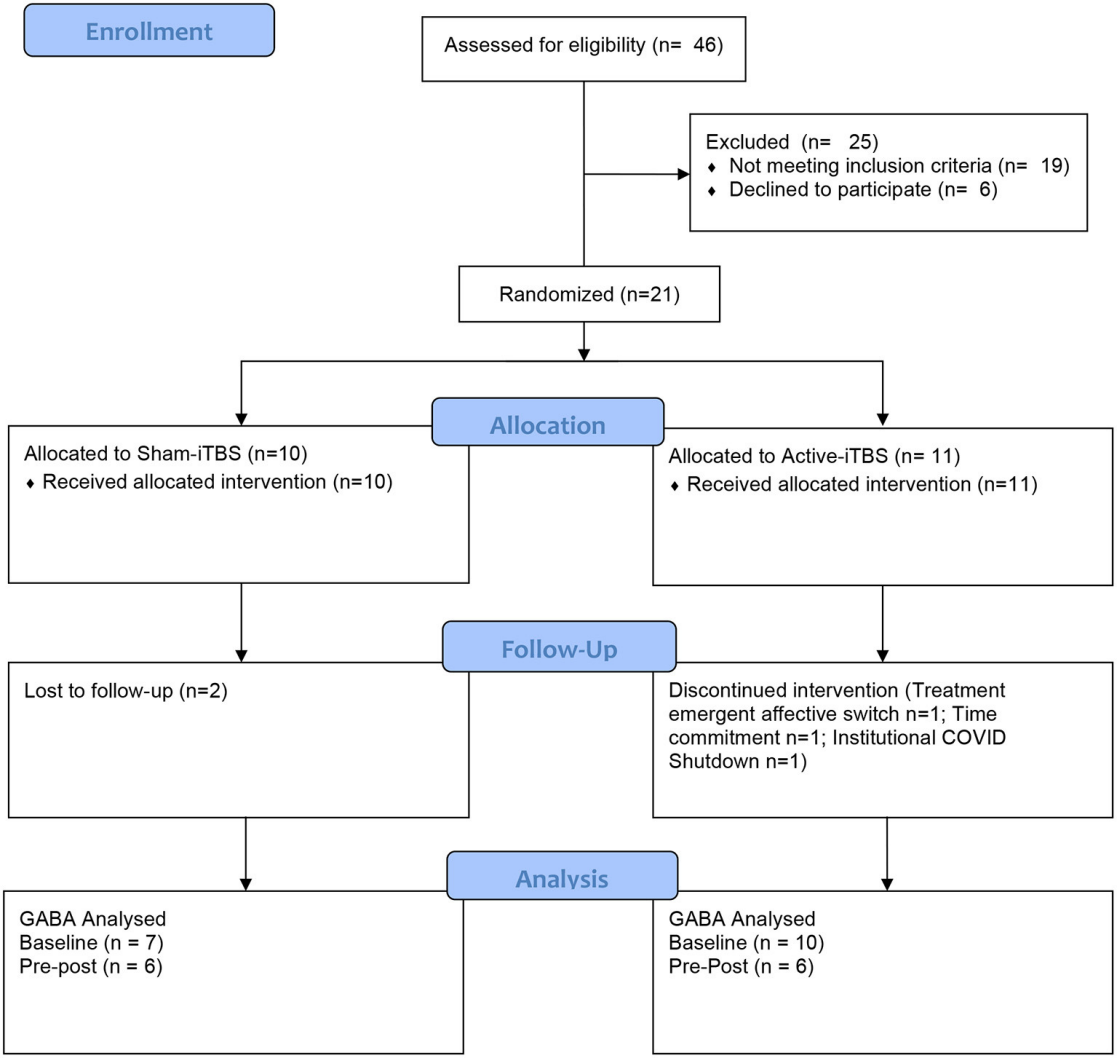
CONSORT flow diagram.

The progress of participants through the University of Calgary site within this multisite study is described in the CONSORT flow diagram (Figure 2). Twenty-one participants were randomized between sham-iTBS (*n* = 10) and active iTBS-rTMS groups (*n* = 11). Three individuals did not tolerate the MRI, and therefore we acquired valid GABA data at baseline from 18 participants (*n* = 7 sham-iTBS and *n* = 11 active-iTBS). Sociodemographic and clinical characteristics were comparable between groups at baseline, and treatment outcomes also did not differ (Table 1).

**Table 1:** Sample characteristics and clinical outcomes.

	**Sham-iTBS**	**Active-iTBS**	**Statistic**	***p***
	***n* (%) or M ± SD**	***n* (%) or M ± SD**		
Age	42.20 ± 13.32	45.27 ± 14.52	*t*(19) = 0.50	0.62
Female sex	5 (50.0%)	5 (45.5%)	χ^2^= 0.04	0.83
Education (years)	16.00 ± 2.16	16.64 ± 3.72	*t*(19) = 0.47	0.64
Diagnosis			χ^2^ = 0.11	0.73
BD1	2 (28.6%)	4 (36.4%)		
BD2	5 (71.4%)	7 (63.6%)		
Medication stratification			χ^2^ = 0.06	0.96
Mood stabilizer	4 (40.0%)	5 (45.5%)		
Atypical antipsychotic	1 (10.0%)	1 (9.1%)		
Mood stabilizer + atypical antipsychotic	5 (50.0%)	5 (45.5%)		
Baseline characteristics				
HRDS-17	20.50 ± 3.30	22.54 ± 3.88	*t*(19) = 1.29	0.21
MADRS	31.60 ± 3.89	32.90 ± 4.30	*t*(19) = 0.72	0.47
CGI-Severity	4.20 ± 0.42	4.70 ± 0.94	*t*(19) = 1.52	0.14
SHAPS	11.00 ± 3.09	10.30 ± 4.00	*t*(18) = −0.43	0.66
Treatment outcomes				
Percent MADRS improvement at 4 weeks	40.33 ± 34.93	24.69 ± 36.48	*t*(15) = −0.90	0.38
Clinical response	3 (33.3%)	1 (12.5%)	χ^2^ = 1.02	0.31
Clinical remission	3 (33.3%)	1 (12.5%)	χ^2^ = 1.02	0.31

mPFC GABA concentrations at baseline were skewed with one notable outlier >3 standard deviations from the mean. When this participant was removed, this data was normally distributed. There were no significant baseline differences in GABA levels between sham-iTBS and active-iTBS groups [*n* = 7 sham-iTBS 2.13 ± 0.36 mmol vs. *n* = 10 active-iTBS 1.96 ± 0.45 mmol *t*_(15)_ = −0.84, *p* = 0.41]. mPFC Glx concentrations were normally distributed and there was a trend toward higher Glx levels at baseline in the sham-iTBS group [*n* = 7 sham-iTBS 11.20 ± 2.53 mol/kg vs. *n* = 10 active-iTBS 9.41 ± 0.94 mmol *t*_(15)_ = −2.06, *p* = 0.057].

**Figure 3:**
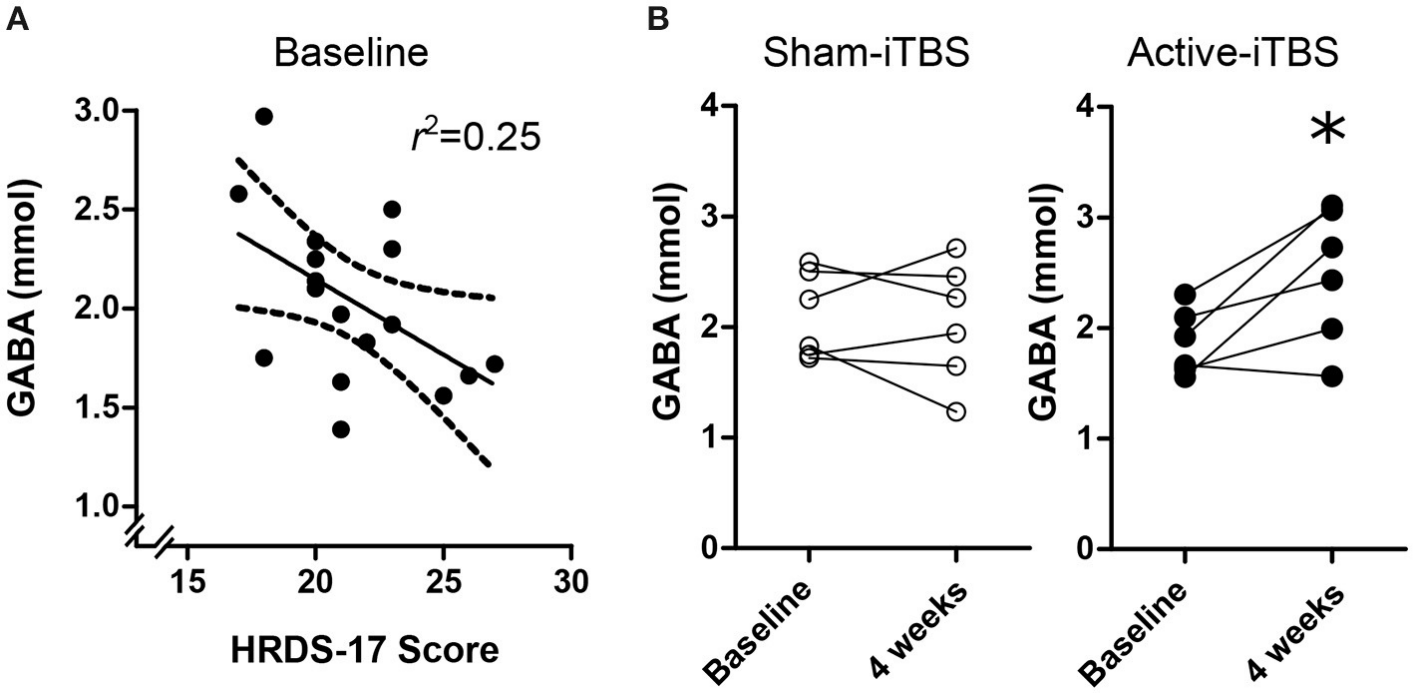
**(A)** Baseline mPFC GABA concentrations were negative associated with HRDS-17 scores. **(B)** Active-iTBS, but not sham-iTBS, was associated with an increase in mPFC GABA concentrations after 4 weeks of treatment. *time interaction *p* < 0.05.

At baseline, there were no significant associations between GABA and SHAPS score [*F*_(1, 15)_ = 0.07, *p* = 0.79], medication regimen [*F*_(2, 16)_ = 0.02, *p* = 0.97], MADRS score [*F*_(1, 16)_ = 2.41, *p* = 0.14], or CGI-severity score [*F*_(1, 15)_ = 1.53, *p* = 0.23], however there was a significant association withHRDS-17 scores [Figure 3A; *F*_(1, 16)_ = 5.18, *p* = 0.038, Standardized *B* = −0.50]. mPFC Glx did not significantly correlate with any clinical characteristic (*p* > 0.18).

At the conclusion of double-blind treatment, paired pre-post MRS data were available for *n* = 6 sham-iTBS and *n* = 6 active-iTBS participants. We observed a significant increase in mPFC GABA in the active-iTBS group, but not the sham-iTBS group [Figure 3B; Time *F*_(1, 10)_ = 4.65, *p* = 0.056, Time ^*^ Group *F*_(1, 10)_ = 6.99, *p* = 0.025]. This significant effect persisted after controlling for percent change in MADRS scores [Time *F*_(1, 9)_ = 0.69, *p* = 0.42, Time ^*^ %MADRS *F*_(1, 9)_ = 1.51, *p* = 0.24, Time ^*^ Group *F*_(1, 9)_ = 8.68, *p* = 0.016]. We found no evidence for mPFC Glx time or treatment group effects [Time *F*_(1, 9)_ = 0.00, *p* = 0.99, Time ^*^ Group *F*_(1, 9)_ = 0.00, *p* = 0.98].

## Discussion

To our knowledge, this is the first study to use GABA-edited MRS to investigate mPFC metabolite changes during TMS therapy in acute bipolar depression. Though preliminary, our data suggests that iTBS results in increased GABA in the mPFC, consistent with previous data in MDD samples for the mPFC (20) and in the DLPFC (42). However, contrary to both of these rTMS studies, and others using pharmacological interventions (19), the GABA effect we observed was dissociated from antidepressant outcomes. We also failed to observe an association between mPFC GABA and anhedonia.

This data is interesting because it confirms a common physiological effect of DLPFC TMS stimulation in different pathologies. Yet, the lack of antidepressant effect in this subsample or in the larger multisite trial from which it is drawn highlights that not all physiological effects of therapeutic TMS are causally associated with improvement in depressive symptoms. Moreover, we did not observe associations between mPFC GABA and clinical features such as anhedonia, a replicated finding in MDD (18). While it is possible that the physiological effect we observe precedes symptomatic improvement, the 4 week open label extension of this trial for non-responders also had a low rate of clinical response (9).

Given the small sample and inference from a null finding, further research is required to determine whether our findings represent a physiological difference between acute bipolar depression and unipolar depression. It is possible that MRS quantified GABA in the mPFC is not as tightly linked to mood state in BD, given that previous studies in BD have not found differences compared to healthy controls in either depression (43) or euthymia (44, 45), and this has also been suggested in meta-analyses (46). Post-mortem staining for GABAergic subpopulations in the anterior cingulate cortex indicate heterogeneity between BD and MDD, however this is principally for calbindin positive neurons and appears layer specific and therefore cannot be resolved with MRS methods (47). Alternatively, concomitant medications indicated for acute and prophylactic treatment in BD may confound MRS GABA measures. Indeed, mood stabilizers modulate GABA (48–50) and lithium modulates Glx (51), and all but one participant was treated with these medications.

## Limitations

Our preliminary data comes from a single site from a randomized controlled trial that was terminated for futility. As a result, the sample size is small, and the availability of paired pre-post MRS data is further reduced. As such, the magnitude of change seen in GABA concentrations in the active-iTBS group may be inflated due to to small sample size bias. Nevertheless, this increase is not observed in the sham-iTBS group, and therefore is likely to be a physiological effect of iTBS. Larger studies are required to more reliably determine the effect size, and independence from clinical improvements in acute bipolar depression. Participants in this trial were required to have stable dosing of an antimanic agent, which resulted in 20 of the 21 participants using medications known to influence GABA (48–50). While an unmedicated sample would resolve this limitation, it represents a safety concern as even with antimanic agents in this sample a treatment emergent affective switch occurred after a single active-iTBS treatment, and another participant experienced a treatment emergent affective switch within eight treatments after transitioning from sham-iTBS to open-label iTBS.

There is an inherent challenge in resolving GABA at 3 T due to its low concentration and overlapped, more abundant metabolites. The use of editing techniques, such as the MEGA-PRESS sequence used in this study, is preferable to the use of short-echo point resolved spectroscopy sequences for measuring GABA, particularly at 3 T (52–54). Nonetheless, there are some limitations and caveats worthy of discussion. Given the low signal and the J-difference editing approach used in MEGA-PRESS, a large voxel and many signal averages are required to obtain high signal-to-noise data for a reliable GABA measurements (53). The voxel used in the present study was 27 cm^3^, placed in the mPFC. This large voxel limits the regional specificity of our results as it encompasses multiple functionally or anatomically distinct structures. Secondly, the measured GABA signal at 3 ppm includes a macromolecule contribution due to the limited specificity of the editing pulses applied at 1.9 ppm (55, 56). Macromolecules are typically considered functionally irrelevant, though the macromolecular signal is variable between subjects (53, 57). Finally, while frequency drift is known to affect efficiency of the editing pulses, the level of macromolecular signal contribution and can cause subtraction artifacts (58), the scanner used in this study is highly stable and no included data showed a frequency drift of >0.1 ppm.

## Conclusion

Our sham-controlled data in acute bipolar depression suggests that iTBS targeting the DLPFC is associated with physiological changes in the mPFC. Unlike previous reports in MDD, in acute bipolar depression mPFC GABA concentrations appear to be dissociated from antidepressant treatment outcomes and anhedonia or have a much smaller effect. Future research is needed to better understand the clinical implications and network level changes associated with neural metabolites in acute bipolar depression.

## Data Availability Statement

The original contributions presented in the study are included in the article/supplementary material, further inquiries can be directed to the corresponding author/s.

## Ethics Statement

The studies involving human participants were reviewed and approved by University of Calgary Conjoint Health Research Ethics Board. The patients/participants provided their written informed consent to participate in this study.

## Author Contributions

Study conception: AM, LY, and AH; Securing study funding: LY; Data collection: JC and AM; Data analyses: CD, MD, AH, and AM; Figure generation: AH and AM; Drafting manuscript: CD; Editing and approval of final manuscript: MD, JC, LY, AH, and AM. All authors contributed to the article and approved the submitted version.

## Conflict of Interest

The authors declare that the research was conducted in the absence of any commercial or financial relationships that could be construed as a potential conflict of interest.

## References

[1] Ferrari AJ Stockings E Khoo JP Erskine HE Degenhardt L Vos T. The prevalence and burden of bipolar disorder: findings from the Global Burden of Disease Study 2013. Bipolar Disord. (2016) 18:440–50. 10.1111/bdi.12423 27566286

[2] Forte A Baldessarini RJ Tondo L Vázquez GH Pompili M Girardi P. Long-term morbidity in bipolar-I, bipolar-II, and unipolar major depressive disorders. J Affect Disord. (2015) 178:71–8. 10.1016/j.jad.2015.02.011 25797049

[3] Yatham LN Kennedy SH Parikh SV Schaffer A Bond DJ Frey BN. Canadian Network for Mood and Anxiety Treatments (CANMAT) and International Society for Bipolar Disorders (ISBD) 2018 guidelines for the management of patients with bipolar disorder. Bipolar Disord. (2018) 20:97–170. 10.1111/bdi.12609 29536616 PMC5947163

[4] McGirr A Karmani S Arsappa R Berlim MT Thirthalli J Muralidharan K. Clinical efficacy and safety of repetitive transcranial magnetic stimulation in acute bipolar depression. World Psychiatry. (2016) 15:85–6. 10.1002/wps.20300 26833619 PMC4780310

[5] Nguyen TD Hieronymus F Lorentzen R McGirr A Østergaard SD. The efficacy of repetitive transcranial magnetic stimulation (rTMS) for bipolar depression: a systematic review and meta-analysis. J Affect Disord. (2021) 279:250–5. 10.1016/j.jad.2020.10.013 33074144

[6] Berlim MT McGirr A Rodrigues Dos Santos N Tremblay S Martins R. Efficacy of theta burst stimulation (TBS) for major depression: an exploratory meta-analysis of randomized and sham-controlled trials. J Psychiatr Res. (2017) 90:102–9. 10.1016/j.jpsychires.2017.02.015 28254709

[7] Li C-T Chen M-H Juan C-H Huang H-H Chen L-F Hsieh J-C. Efficacy of prefrontal theta-burst stimulation in refractory depression: a randomized sham-controlled study. Brain J Neurol. (2014) 137:2088–98. 10.1093/brain/awu109 24817188

[8] Bulteau S Beynel L Marendaz C Dall'Igna G Peré M Harquel S. Twice-daily neuronavigated intermittent theta burst stimulation for bipolar depression: a randomized Sham-controlled pilot study. Neurophysiol Clin Clin Neurophysiol. (2019) 49:371–5. 10.1016/j.neucli.2019.10.002 31761447

[9] McGirr A Vila-Rodriguez F Cole J Torres IJ Arumugham SS Keramatian K. Efficacy of active vs Sham intermittent theta burst transcranial magnetic stimulation for patients with bipolar depression: a randomized clinical trial. JAMA Netw Open. (2021) 4:e210963. 10.1001/jamanetworkopen.2021.0963 33710288 PMC7955269

[10] Duman RS Sanacora G Krystal JH. Altered connectivity in depression: GABA and glutamate neurotransmitter deficits and reversal by novel treatments. Neuron. (2019) 102:75–90. 10.1016/j.neuron.2019.03.013 30946828 PMC6450409

[11] Schür RR Draisma LWR Wijnen JP Boks MP Koevoets MGJC Joëls M. Brain GABA levels across psychiatric disorders: a systematic literature review and meta-analysis of 1H-MRS studies. Hum Brain Mapp. (2016) 37:3337–52. 10.1002/hbm.23244 27145016 PMC6867515

[12] Godfrey KEM Gardner AC Kwon S Chea W Muthukumaraswamy SD. Differences in excitatory and inhibitory neurotransmitter levels between depressed patients and healthy controls: a systematic review and meta-analysis. J Psychiatr Res. (2018) 105:33–44. 10.1016/j.jpsychires.2018.08.015 30144668

[13] Hasler G van der Veen JW Tumonis T Meyers N Shen J Drevets WC. Reduced prefrontal glutamate/glutamine and γ-aminobutyric acid levels in major depression determined using proton magnetic resonance spectroscopy. Arch Gen Psychiatry. (2007) 64:193–200. 10.1001/archpsyc.64.2.193 17283286

[14] Krystal JH Sanacora G Blumberg H Anand A Charney DS Marek G. Glutamate and GABA systems as targets for novel antidepressant and mood-stabilizing treatments. Mol Psychiatry. (2002) 7:S71–80. 10.1038/sj.mp.4001021 11986998

[15] Price RB Shungu DC Mao X Nestadt P Kelly C Collins KA. Amino acid neurotransmitters assessed by proton magnetic resonance spectroscopy: relationship to treatment resistance in major depressive disorder. Biol Psychiatry. (2009) 65:792–800. 10.1016/j.biopsych.2008.10.025 19058788 PMC2934870

[16] Colic L von Düring F Denzel D Demenescu LR Lord AR Martens L. Rostral anterior cingulate glutamine/glutamate disbalance in major depressive disorder depends on symptom severity. Biol Psychiatry Cogn Neurosci Neuroimaging. (2019) 4:1049–58. 10.1016/j.bpsc.2019.04.003 31202822

[17] Gabbay V Bradley KA Mao X Ostrover R Kang G Shungu DC. Anterior cingulate cortex γ-aminobutyric acid deficits in youth with depression. Transl Psychiatry. (2017) 7:e1216. 10.1038/tp.2017.187 28892070 PMC5611750

[18] Gabbay V Mao X Klein RG Ely BA Babb JS Panzer AM. Anterior cingulate cortex γ-aminobutyric acid in depressed adolescents: relationship to anhedonia. Arch Gen Psychiatry. (2012) 69:139–49. 10.1001/archgenpsychiatry.2011.131 21969419 PMC3711232

[19] Brennan BP Admon R Perriello C LaFlamme EM Athey AJ Pizzagalli DA. Acute change in anterior cingulate cortex GABA, but not glutamine/glutamate, mediates antidepressant response to citalopram. Psychiatry Res Neuroimaging. (2017) 269:9–16. 10.1016/j.pscychresns.2017.08.009 28892734 PMC5642118

[20] Dubin MJ Mao X Banerjee S Goodman Z Lapidus KAB Kang G. Elevated prefrontal cortex GABA in patients with major depressive disorder after TMS treatment measured with proton magnetic resonance spectroscopy. J Psychiatry Neurosci. (2016) 41:E37–45. 10.1503/jpn.150223 26900793 PMC4853214

[21] Iwabuchi SJ Raschke F Auer DP Liddle PF Lankappa ST Palaniyappan L. Targeted transcranial theta-burst stimulation alters fronto-insular network and prefrontal GABA. NeuroImage. (2017) 146:395–403. 10.1016/j.neuroimage.2016.09.043 27651067

[22] Hadas I Sun Y Lioumis P Zomorrodi R Jones B Voineskos D. Association of repetitive transcranial magnetic stimulation treatment with subgenual cingulate hyperactivity in patients with major depressive disorder: a secondary analysis of a randomized clinical trial. JAMA Netw Open. (2019) 2:e195578. 10.1001/jamanetworkopen.2019.5578 31167023 PMC6551850

[23] Boes AD Uitermarkt BD Albazron FM Lan MJ Liston C Pascual-Leone A. Rostral anterior cingulate cortex is a structural correlate of repetitive TMS treatment response in depression. Brain Stimulat. (2018) 11:575–81. 10.1016/j.brs.2018.01.029 29454551 PMC6136654

[24] Fox MD Buckner RL White MP Greicius MD Pascual-Leone A. Efficacy of transcranial magnetic stimulation targets for depression is related to intrinsic functional connectivity with the subgenual cingulate. Biol Psychiatry. (2012) 72:595–603. 10.1016/j.biopsych.2012.04.028 22658708 PMC4120275

[25] Weigand A Horn A Caballero R Cooke D Stern AP Taylor SF. Prospective Validation that subgenual connectivity predicts antidepressant efficacy of transcranial magnetic stimulation sites. Biol Psychiatry. (2018) 84:28–37. 10.1016/j.biopsych.2017.10.028 29274805 PMC6091227

[26] Northoff G Walter M Schulte RF Beck J Dydak U Henning A. GABA concentrations in the human anterior cingulate cortex predict negative BOLD responses in fMRI. Nat Neurosci. (2007) 10:1515–7. 10.1038/nn2001 17982452

[27] Hamilton M. A rating scale for depression. J Neurol Neurosurg Psychiatry. (1960) 23:56–62. 10.1136/jnnp.23.1.56 14399272 PMC495331

[28] Blumberger DM Vila-Rodriguez F Thorpe KE Feffer K Noda Y Giacobbe P. Effectiveness of theta burst versus high-frequency repetitive transcranial magnetic stimulation in patients with depression (THREE-D): a randomised non-inferiority trial. Lancet Lond Engl. (2018) 391:1683–92. 10.1016/S0140-6736(18)30295-2 29726344

[29] Beam W Borckardt JJ Reeves ST George MS. An efficient and accurate new method for locating the F3 position for prefrontal TMS applications. Brain Stimulat. (2009) 2:50–4. 10.1016/j.brs.2008.09.006 20539835 PMC2882797

[30] Montgomery SA Asberg M. A new depression scale designed to be sensitive to change. Br J Psychiatry J Ment Sci. (1979) 134:382–9. 10.1192/bjp.134.4.382 444788

[31] Young RC Biggs JT Ziegler VE Meyer DA. A rating scale for mania: reliability, validity and sensitivity. Br J Psychiatry. (1978) 133:429–35. 10.1192/bjp.133.5.429 728692

[32] Guy W. ECDEU Assessment Manual for Psychopharmacology. U.S. Department of Health, Education, and Welfare, Public Health Service, Alcohol, Drug Abuse, and Mental Health Administration, National Institute of Mental Health, Psychopharmacology Research Branch, Division of Extramural Research Programs (1976).

[33] Snaith RP Hamilton M Morley S Humayan A Hargreaves D Trigwell P. A scale for the assessment of hedonic tone the Snaith-Hamilton pleasure scale. Br J Psychiatry J Ment Sci. (1995) 167:99–03. 10.1192/bjp.167.1.99 7551619

[34] Harris AD Saleh MG Edden RAE. Edited 1 H magnetic resonance spectroscopy *in vivo*: methods and metabolites. Magn Reson Med. (2017) 77:1377–89. 10.1002/mrm.26619 28150876 PMC5352552

[35] Edden RAE Puts NAJ Harris AD Barker PB Evans CJ. Gannet: a batch-processing tool for the quantitative analysis of gamma-aminobutyric acid–edited MR spectroscopy spectra. J Magn Reson Imaging. (2014) 40:1445–52. 10.1002/jmri.24478 25548816 PMC4280680

[36] Gasparovic C Song T Devier D Bockholt HJ Caprihan A Mullins PG. Use of tissue water as a concentration reference for proton spectroscopic imaging. Magn Reson Med. (2006) 55:1219–26. 10.1002/mrm.20901 16688703

[37] Near J Harris AD Juchem C Kreis R Marjańska M Öz G. Preprocessing, analysis and quantification in single-voxel magnetic resonance spectroscopy: experts' consensus recommendations. NMR Biomed. (2020) 34:e4257. 10.1002/nbm.4257 32084297 PMC7442593

[38] Harris AD Puts NAJ Edden RAE. Tissue correction for GABA-edited MRS: considerations of voxel composition, tissue segmentation, and tissue relaxations. J Magn Reson Imaging. (2015) 42:1431–40. 10.1002/jmri.24903 26172043 PMC4615266

[39] Jenkinson M Smith S. A global optimisation method for robust affine registration of brain images. Med Image Anal. (2001) 5:143–56. 10.1016/s1361-8415(01)00036-6 11516708

[40] Jenkinson M Bannister P Brady M Smith S. Improved optimization for the robust and accurate linear registration and motion correction of brain images. NeuroImage. (2002) 17:825–41. 10.1016/s1053-8119(02)91132-8 12377157

[41] Tukey JW. Exploratory Data Analysis. Reading, MA: Addison-Wesley Pub. Co. (1977).

[42] Levitt JG Kalender G O'Neill J Diaz JP Cook IA Ginder N. Dorsolateral prefrontal γ-aminobutyric acid in patients with treatment-resistant depression after transcranial magnetic stimulation measured with magnetic resonance spectroscopy. J Psychiatry Neurosci. (2019) 44:386–94. 10.1503/jpn.180230 31199104 PMC6821508

[43] Soeiro-de-Souza MG Henning A Machado-Vieira R Moreno RA Pastorello BF da Costa Leite C. Anterior cingulate Glutamate–Glutamine cycle metabolites are altered in euthymic bipolar I disorder. Eur Neuropsychopharmacol. (2015) 25:2221–9. 10.1016/j.euroneuro.2015.09.020 26476706

[44] Huber RS Kondo DG Shi X-F Prescot AP Clark E Renshaw PF. Relationship of executive functioning deficits to N-acetyl aspartate (NAA) and gamma-aminobutyric acid (GABA) in youth with bipolar disorder. J Affect Disord. (2018) 225:71–8. 10.1016/j.jad.2017.07.052 28800423

[45] Prisciandaro JJ Tolliver BK Prescot AP Brenner HM Renshaw PF Brown TR. Unique prefrontal GABA and glutamate disturbances in co-occurring bipolar disorder and alcohol dependence. Transl Psychiatry. (2017) 7:e1163. 10.1038/tp.2017.141 28675386 PMC5538121

[46] Scotti-Muzzi E Umla-Runge K Soeiro-de-Souza MG. Anterior cingulate cortex neurometabolites in bipolar disorder are influenced by mood state and medication: a meta-analysis of 1H-MRS studies. Eur Neuropsychopharmacol. (2021). 10.1016/j.euroneuro.2021.01.096 33581932

[47] Cotter D Landau S Beasley C Stevenson R Chana G MacMillan L. The density and spatial distribution of gabaergic neurons, labelled using calcium binding proteins, in the anterior cingulate cortex in major depressive disorder, bipolar disorder, and schizophrenia. Biol Psychiatry. (2002) 51:377–86. 10.1016/S0006-3223(01)01243-4 11904132

[48] Berrettini WH Nurnberger JI Hare T Gershon ES Post RM. Plasma and CSF GABA in affective illness. Br J Psychiatry. (1982) 141:483–7. 10.1192/bjp.141.5.483 7150885

[49] Löscher W. Anticonvulsant and biochemical effects of inhibitors of GABA aminotransferase and valproic acid during subchronic treatment in mice. Biochem Pharmacol. (1982) 31:837–42. 10.1016/0006-2952(82)90471-3 6805473

[50] Romeo B Choucha W Fossati P Rotge JY. Meta-analysis of central and peripheral γ-aminobutyric acid levels in patients with unipolar and bipolar depression. J Psychiatry Neurosci. (2018) 43:58–66. 10.1503/jpn.160228 29252166 PMC5747536

[51] Machado-Vieira R Gattaz WF Zanetti MV De Sousa RT Carvalho AF Soeiro-de-Souza MG. A longitudinal (6-week) 3T 1H-MRS study on the effects of lithium treatment on anterior cingulate cortex metabolites in bipolar depression. Eur Neuropsychopharmacol. (2015) 25:2311–7. 10.1016/j.euroneuro.2015.08.023 26428274

[52] Mescher M Merkle H Kirsch J Garwood M Gruetter R. Simultaneous *in vivo* spectral editing and water suppression. NMR Biomed. (1998) 11:266–72. 10.1002/(sici)1099-1492(199810)11:6<266::aid-nbm530>3.0.co;2-j 9802468

[53] Mullins PG McGonigle DJ O'Gorman RL Puts NAJ Vidyasagar R Evans CJ. Current practice in the use of MEGA-PRESS spectroscopy for the detection of GABA. NeuroImage. (2014) 86:43–52. 10.1016/j.neuroimage.2012.12.004 23246994 PMC3825742

[54] Rothman DL Petroff OA Behar KL Mattson RH. Localized 1H NMR measurements of gamma-aminobutyric acid in human brain *in vivo*. Proc Natl Acad Sci U S A. (1993) 90:5662–6. 10.1073/pnas.90.12.5662 8516315 PMC46781

[55] Henry PG Dautry C Hantraye P Bloch G. Brain GABA editing without macromolecule contamination. Magn Reson Med. (2001) 45:517–20. 10.1002/1522-2594(200103)45:3<517::aid-mrm1068>3.0.co;2-6 11241712

[56] Behar KL Rothman DL Spencer DD Petroff OA. Analysis of macromolecule resonances in 1H NMR spectra of human brain. Magn Reson Med. (1994) 32:294–302. 10.1002/mrm.1910320304 7984061

[57] Harris AD Puts NAJ Barker PB Edden RAE. Spectral-editing measurements of GABA in the human brain with and without macromolecule suppression. Magn Reson Med. (2015) 74:1523–9. 10.1002/mrm.25549 25521836 PMC4470877

[58] Harris AD Glaubitz B Near J John Evans C Puts NAJ Schmidt-Wilcke T. Impact of frequency drift on gamma-aminobutyric acid-edited MR spectroscopy. Magn Reson Med. (2014) 72:941–8. 10.1002/mrm.25009 24407931 PMC4017007

